# Mycophenolate mofetil for immune checkpoint inhibitor‐related hepatotoxicity relapsing during dose reduction of corticosteroid: A report of two cases and literature review

**DOI:** 10.1002/cnr2.1624

**Published:** 2022-05-16

**Authors:** Masayuki Ueno, Hiroyuki Takabatake, Ayako Hata, Takahisa Kayahara, Youichi Morimoto, Kenji Notohara, Motowo Mizuno

**Affiliations:** ^1^ Department of Gastroenterology and Hepatology Kurashiki Central Hospital Okayama Japan; ^2^ Department of Gastroenterology and Hepatology, Graduate School of Medicine Kyoto University Kyoto Japan; ^3^ Department of Anatomic Pathology Kurashiki Central Hospital Okayama Japan

**Keywords:** durvalumab, immune checkpoint inhibitor, immune checkpoint inhibitor hepatotoxicity, immune checkpoint inhibitor‐related adverse event, mycophenolate mofetil, pembrolizumab

## Abstract

**Background:**

Immune checkpoint inhibitors (ICIs) sometimes cause immune‐related liver injury, which can lead to cessation of treatment, hospitalization, and even mortality. Although high‐dose corticosteroids are usually effective in treatment of ICI‐related liver injury, one fifth of affected patients require additional immunosuppressive therapy. It remains uncertain how best to treat ICI‐related liver injury that relapses under corticosteroid therapy after temporary remission.

**Case:**

Here we report two cases of ICI‐related liver injury successfully treated with mycophenolate mofetil (MMF). In the first case, a 74‐year‐old man with stage IIIA lung cancer underwent curative chemoradiotherapy. After the second infusion of durvalumab, grade 3 ICI‐related liver injury (mixed pattern) developed. In the second case, a 46‐year‐old man with stage IVB lung cancer received pembrolizumab‐containing chemotherapy. After the first cycle, grade 2 ICI‐related hepatitis developed. In the both cases, liver injury improved with high‐dose prednisolone but relapsed during tapering of the drug. After liver biopsy was performed to confirm the diagnosis of ICI‐related liver injury, MMF (2000 mg/day) was added. MMF was effective for both patients and permitted discontinuation or reduction of prednisolone.

**Conclusion:**

MMF appears to be an appropriate treatment option for ICI‐related liver injury that respond to high‐dose corticosteroids but relapse during steroid tapering.

## 1 INTRODUCTION

Immune checkpoint inhibitors (ICIs) are increasingly used for treatment of various cancers.^1^, ^2^ ICIs provide an antitumor effect by blocking regulatory immune checkpoint molecules and enhancing T cell activity.^3^ However, the inhibitors sometimes disturb the immune system and cause immune‐related adverse events (irAEs),^4^ which can lead to cessation of treatment, hospitalization, long‐term symptoms, and even mortality.^5^ ICI‐related hepatotoxicity is one of the commonest irAEs; it occurs in 0.7%–1.8% of patients receiving anti‐programmed death‐1 (PD‐1)/programmed death‐ligand 1 (PD‐L1) agents and in 4%–11% of patients receiving anti‐cytotoxic T‐lymphocyte‐associated protein‐4 agents.^6^ Although high‐dose corticosteroids are usually effective in treatment of ICI‐related liver injury, one fifth of affected patients require additional immunosuppressive therapy.^7^ Mycophenolate mofetil (MMF) is one of the most often used drugs for treatment of refractory irAE.^8^, ^9^ However, evidence is lacking on how best to treat ICI‐related liver injury that relapses under corticosteroid therapy after temporary remission; here, we report two cases illustrating the efficacy of MMF for such patients. The use of MMF for ICI‐related liver toxicity is not covered by health insurance in Japan; thus, we used MMF with permission by the Department of Evaluation for Unapproved and Off‐label Uses of Kurashiki Central Hospital (No. 90183). This case report was prepared according to CARE Guidelines.^10^

## 2 CASE 1

**FIGURE 1:**
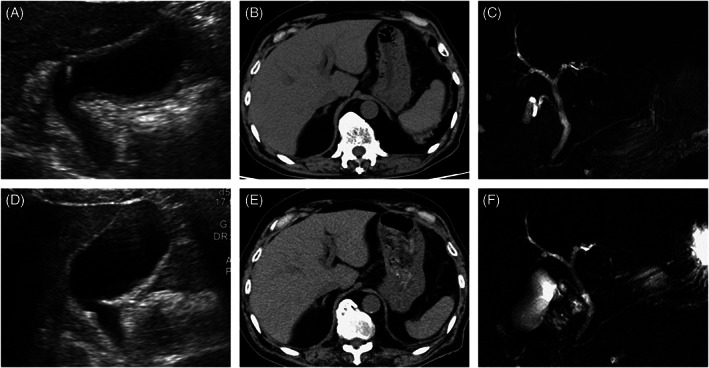
Radiological findings of Case 1: (A–C) are figures at the onset of liver injury: (A) ultrasonography shows diffuse thickening of the gallbladder wall; (B, C) no abnormal findings are present in CT and MRI; (D–F) are figures at the relapse of liver injury; no abnormalities are present in ultrasonography, CT, and MRI

**FIGURE 2:**
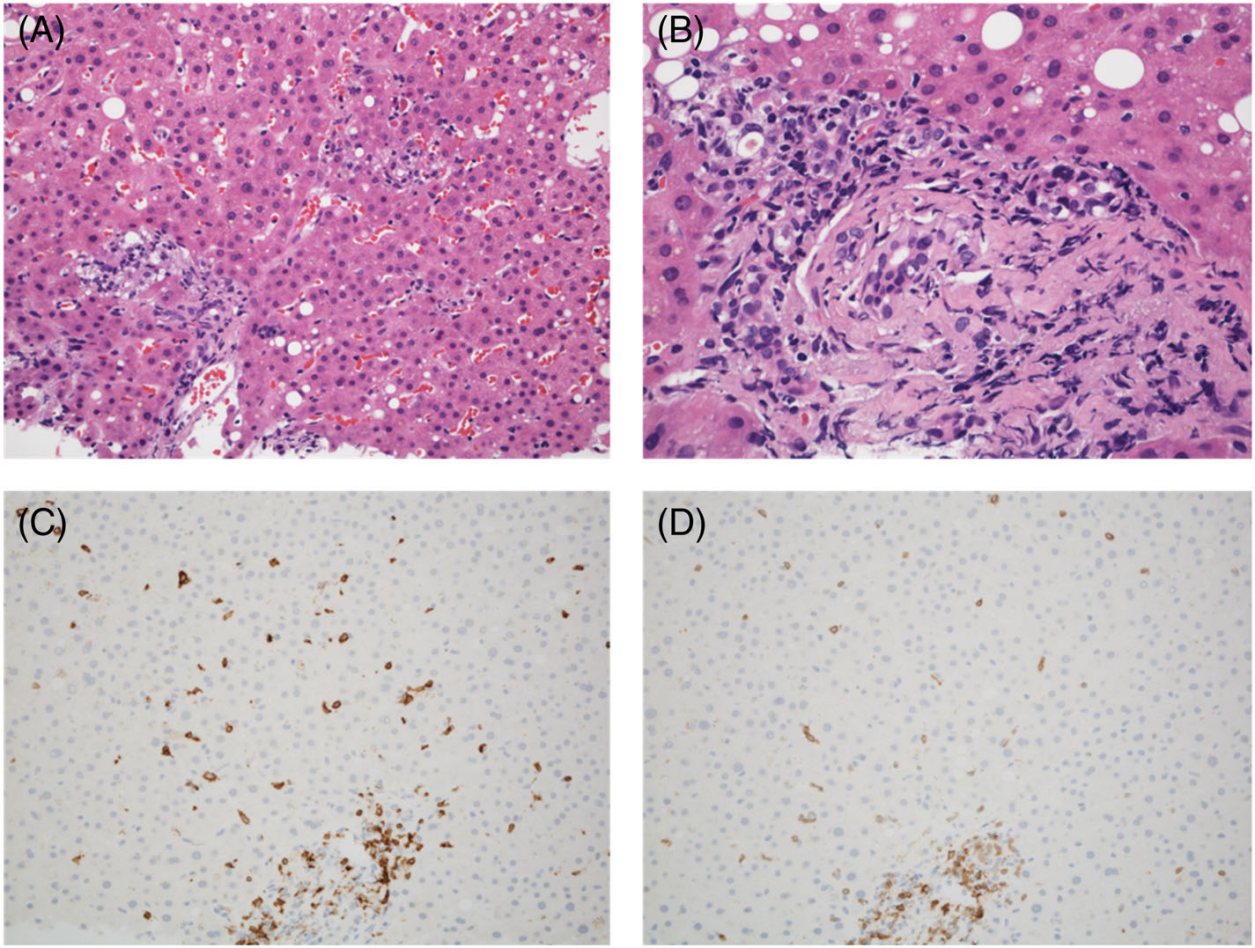
Histological findings of Case 1. (A) Focal necrosis is present in the lobular parenchyma (hematoxylin and eosin staining, ×20). (B) A damaged interlobular bile duct has an irregular configuration and intraepithelial inflammatory cells. The surrounding infiltrate is rich in lymphocytes (hematoxylin and eosin staining, ×40). (C) CD8‐positive cells are present in the portal area and the lobular parenchyma (CD8 staining, ×20). (D) Distribution of CD3‐positive cells similar to that of CD8‐positive cells (CD3 staining, ×20)

**FIGURE 3:**
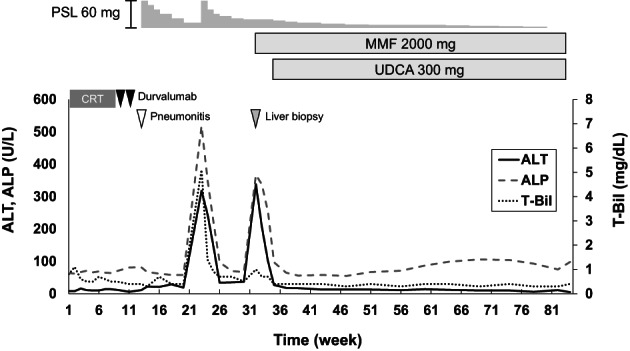
Clinical course of Case 1. CRT, chemoradiotherapy; MMF, mycophenolate mofetil; PSL, prednisolone; UDCA, ursodeoxycholic acid

A 74‐year‐old man with 30 pack years of smoking had a diagnosis of squamous cell lung cancer. He underwent right upper lobe resection with lymph‐node dissection; the TNM status was T1bN0M0 (stage IA2). Fifteen months later, computed tomography (CT) revealed another tumor in the right middle lobe and enlarged lymph nodes in the right hilum. Squamous cell lung cancer was diagnosed again, with endobronchial ultrasound‐guided biopsy (cT1bN2M0, stage IIIA). Additional lung resection was not feasible because of his poor respiratory function. Thus, he received curative chemoradiotherapy consisting of radiotherapy (60 Gy), carboplatin, and paclitaxel, followed by durvalumab (anti‐PD‐L1 drug). After the second cycle of durvalumab, CT revealed ground‐glass opacities in both lungs. ICI‐induced or radiation‐induced pneumonitis was suspected, and high‐dose prednisolone (60 mg [1 mg/kg]/day) was started. After the dose of prednisolone was reduced to 10 mg/day, grade 3 liver injury developed: total bilirubin (T‐Bil) 5.1 [normal range, 0.4–1.5] mg/dl, aspartate aminotransferase (AST) 156 [13–30] U/L, alanine transaminase (ALT) 318 [10–42] U/L, and alkaline phosphatase (ALP) 518 [38–113] U/L, with a normal prothrombin time. An ultrasound scan revealed diffuse thickening of the gallbladder wall (Figure 1A), but no abnormality was evident in the liver and the biliary tract on CT or magnetic resonance imaging (MRI) (Figure 1B,C). The dose of prednisolone was increased to 60 mg/day, which resulted in improved liver biochemistry values, but they deteriorated after the dose was reduced to 17.5 mg/day: T‐Bil 1.0 mg/dl, AST 132 U/L, ALT 338 U/L, and ALP 367 U/L. Thickening of the gallbladder wall was not present on ultrasonography (Figure 1D), and no abnormalities were present on CT and MRI (Figure 1E,F). Percutaneous liver biopsy revealed inflammatory cells, mainly CD8‐positive T cells, infiltrating the portal area and the lobular parenchyma, and irregular configuration of the bile ducts (Figure 2). Thus, ICI‐related liver injury (mixed pattern, cholangitis predominant) was diagnosed. We added MMF 2000 mg/day, without changing the dose of prednisolone. Three weeks later, liver function tests normalized. One year later, prednisolone was discontinued without relapse of biochemical tests (Figure 3).

## 3 CASE 2

**FIGURE 4:**
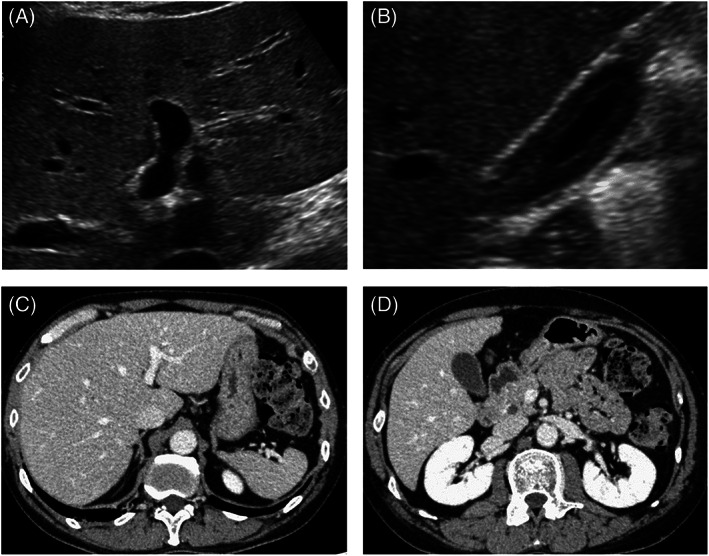
Radiological findings of Case 2. (A) Ultrasonography reveals no apparent abnormality in the liver. (B) Diffuse thickening of the gallbladder wall is present. (C and D) Contrast‐enhanced CT reveals no abnormal findings

**FIGURE 5:**
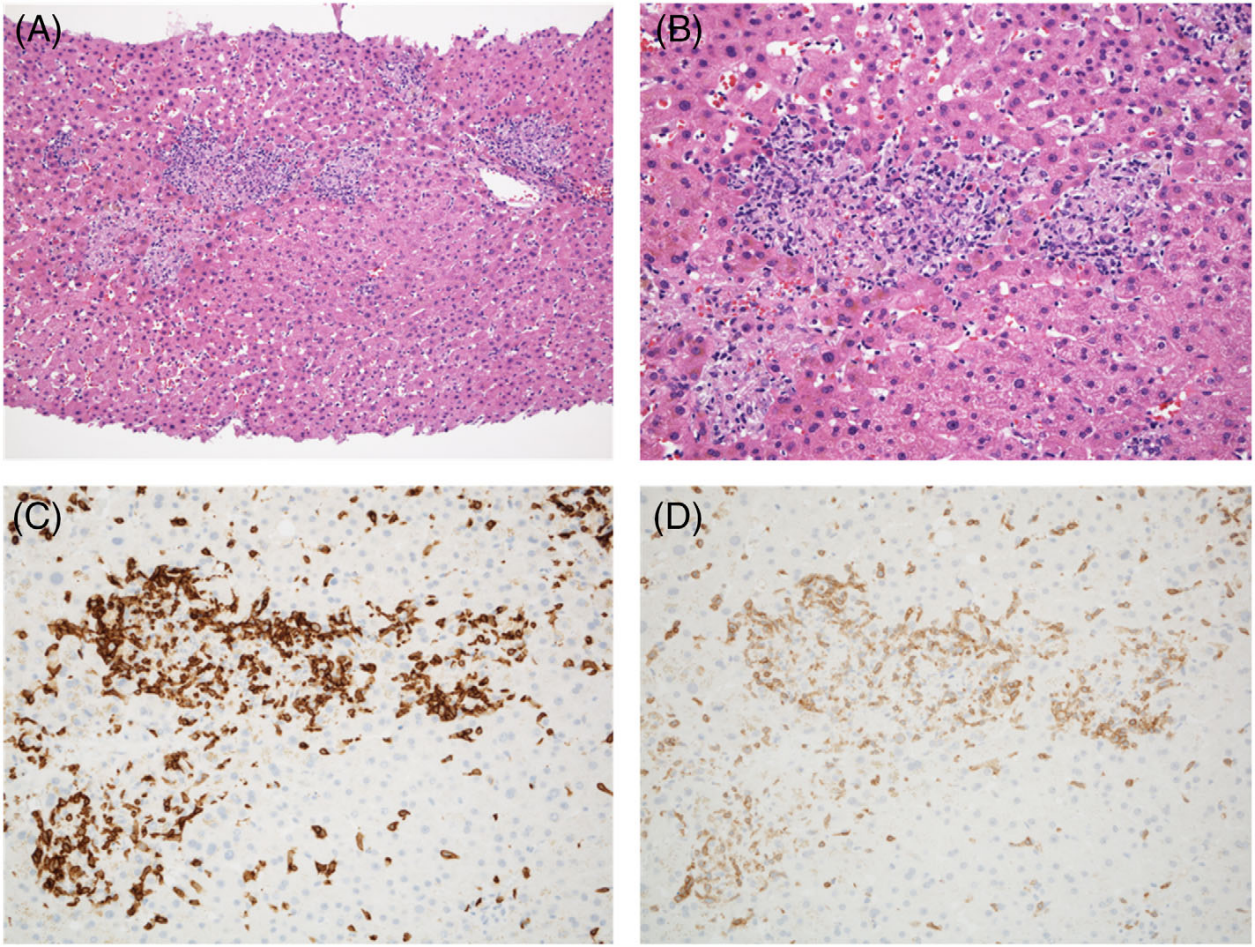
Histological findings of Case 2. (A and B) Panlobular inflammation and necrosis are present. Infiltrating cells are mainly lymphocytes and macrophages (hematoxylin and eosin staining; A: ×10, B: ×20). (C) Many CD8‐positive cells are presents in the inflamed area (CD8 staining, ×20). (D) Distribution of CD3‐positive cells is like that of CD8‐positive cells (CD3 staining, ×20)

**FIGURE 6:**
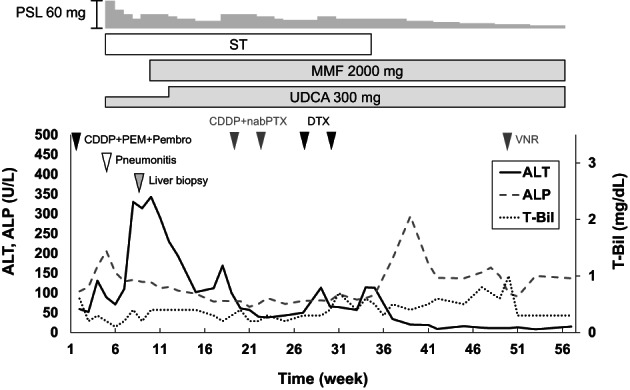
Clinical course of Case 2. CDDP, cisplatin; DTX, docetaxel; MMF, mycophenolate mofetil; nabPTX, nab‐paclitaxel; PEM, pemetrexed; Pembro, pembrolizumab; PSL, prednisolone; ST, sulfamethoxazole‐trimethoprim; UDCA, ursodeoxycholic acid; VNR, vinorelbine

A 46‐year‐old man with no significant past medical history presented with bone pain in the lower limbs. He had 30 pack years of smoking and 40 g/day alcohol consumption. Adenocarcinoma in the right lung and multiple bone metastasis were diagnosed by CT and endobronchial ultrasound‐guided needle aspiration (cT2aN2M1c, stage IVB). Immunohistochemistry revealed staining for PD‐L1, whereas driver mutations, such as epidermal growth factor receptor, anaplastic lymphoma kinase, c‐ros oncogene 1, v‐raf murine sarcoma viral oncogene homolog B1, and mesenchymal–epithelial transition factor were negative. He received combination chemotherapy consisting of cisplatin, pemetrexed and pembrolizumab (anti‐PD‐1 drug). Three weeks after the first infusion of chemotherapy, liver tests were abnormal: T‐Bil 0.3 mg/dl, AST 61 U/L, ALT 131 U/L, and ALP 167 U/L. At the same time, ICI‐related pneumonitis was diagnosed. High‐dose prednisolone (60 mg/day [1 mg/kg/day]) and ursodeoxycholic acid (150 mg/day) were started for treatment of the irAEs. Sulfamethoxazole‐trimethoprim (400 mg/80 mg, triweekly) was also started to prevent opportunistic infection. Liver biochemistry tests improved once but worsened after the prednisolone dose was reduced to 20 mg/day: T‐Bil 0.4 mg/dl, AST 179 U/L, ALT 330 U/L, and ALP 133 U/L. As in the first patient, diffuse thickening of the gallbladder wall was the only abnormal finding on imaging examinations (Figure 4). Percutaneous liver biopsy revealed panlobular inflammation and necrosis, with rich infiltrate of CD8‐positive T cells (Figure 5); the diagnosis of ICI‐related hepatitis was made. We added MMF (2000 mg/day) and increased the dose of ursodeoxycholic acid to 300 mg/day. His liver tests gradually improved. Chemotherapy with cisplatin plus nab‐paclitaxel (second line), docetaxel (third line) and vinorelbine (fourth line) was given. Six months after starting MMF, liver injury worsened but again improved with cessation of sulfamethoxazole‐trimethoprim. Increased ALP values were thought due to progression of bone metastases. At the time of this writing, the dose of prednisolone has been reduced to 5 mg/day without relapse of evident liver injury (Figure 6).

## 4 DISCUSSION

Here we described two cases of ICI‐related liver injury that improved with high‐dose prednisolone once, but relapse occurred during prednisolone tapering. Off‐label use of MMF was effective for both patients and permitted discontinuation or reduction of prednisolone. Although clinical guidelines recommend prescribing MMF when ICI‐related liver injury does not improve with corticosteroids,^8^, ^9^ the reports on usage of MMF for irAEs are few. We have summarized 15 articles reporting the clinical course of hepatic irAEs treated with MMF (Table 1).^11^, ^12^, ^13^, ^14^, ^15^, ^16^, ^17^, ^18^, ^19^, ^20^, ^21^, ^22^, ^23^, ^24^, ^25^ The types of liver injury were classified as hepatitis, cholangitis or mixed pattern based on pathological findings or, if unavailable, R‐ratios at diagnosis of immune‐related liver injury.^26^ Most patients were primarily refractory to steroids; the efficacy rate of MMF in them was less than half. Especially, mixed‐pattern liver injury appeared tend to be refractory to MMF. On the other hand, one patient received MMF for relapsing liver injury during steroid tapering and she had a good response to MMF.^17^ The patient was initially treated with prednisolone of 80 mg/day, then 40 mg/day, with subsequent dose reductions by 5–10 mg. Liver injury improved at first, but relapsed when the prednisolone dose was reduced to 25–30 mg/day. After addition of MMF of 2000 mg/day, liver enzymes normalized in 2 weeks. Taken together with our cases, treatment with MMF appears to be a good option for ICI‐related liver injury relapsing during steroid tapering.

**TABLE 1:** Previous reports of immune‐related liver injury treated with mycophenolate mofetil

Age	Sex	Cancer type	ICI regimen	Grade of liver injury	Type of liver injury	Initial treatment	Response to initial therapy	Reason for adding MMF	Efficacy of MMF	Adverse events of MMF	Reference
50	F	Melanoma	Ipilimumab	4	Hepatitis	mPSL	No	Steroid‐refractory	Yes	None	[11]
59	M	Melanoma	Nivolumab followed by ipilimumab	4	Hepatitis	mPSL	Partial	Steroid‐refractory	Yes	None	[12]
49	F	Melanoma	Ipilimumab + nivolumab	4	Hepatitis	mPSL	No	Steroid‐refractory	No	None	[13]
49	F	Melanoma	Pembrolizumab	4	Mixed	PSL	No	Steroid‐refractory	No	Neutropenia	[14]
76	M	Epithelioid mesothelioma	Pembrolizumab	4	Mixed	mPSL	No	Steroid‐refractory	No	Lymphopenia	[14]
57	F	Melanoma	Anti‐PD‐1 drug	4	Hepatitis	mPSL	Partial	Steroid‐refractory	No	None	[15]
81	F	NSCLC	Nivolumab	4	Cholangitis	mPSL	Partial	Steroid‐refractory	Yes	Abscess	[16]
55	M	NSCLC	Nivolumab	4	Cholangitis	mPSL + MMF	Yes	Based on clinicians' experience	Yes	None	[16]
53	F	CHC	Pembrolizumab	4	Hepatitis	PSL	Temporaly	Relapse during steroid tapepring	Yes	None	[17]
50	M	Laryngeal cancer	Nivolumab	3	Hepatitis	mPSL	No	Steroid‐refractory	Yes	None	[18]
70s	M	Melanoma	Nivolumab followed by ipilimumab	4	Hepatitis	mPSL	No	Steroid‐refractory	No	None	[19]
69	M	NSCLC	Durvalumab	3	Mixed	mPSL	No	Steroid‐refractory	No	None	[20]
64	M	NSCLC	Nivolumab	3	Mixed	PSL	Partial	Steroid‐refractory	No	None	[21]
75	M	NSCLC	Nivolumab	3	Cholangitis	PSL + UDCA	Partial	Steroid‐refractory	No	None	[22]
46	M	Melanoma	Nivolumab	4	Hepatitis	mPSL	No	Steroid‐refractory	No	None	[23]
65	M	Esophageal cancer	Camrelizumab	3	Mixed	mPSL + UDCA	No	Steroid‐refractory	No	None	[24]
75	M	Melanoma	Ipilimumab + nivolumab	3	Hepatitis	mPSL	Partial	Steroid‐refractory	Partial	None	[25]

Abbreviations: CHC, combined hepatocellular‐cholangiocarcinoma; F, female; ICI, immune‐checkpoint inhibitor; M, male; MMF, mycophenolate mofetil; mPSL, methylprednisolone; NSCLC, non‐small cell lung cancer; PD‐1, programmed cell death‐1; PSL, prednisolone; UDCA, ursodeoxycholic acid.

ICI‐related hepatitis has features resembling autoimmune hepatitis.^27^ Therefore, referring to the reported response of autoimmune hepatitis to MMF may be helpful when using the drug for ICI‐related liver injury. In a prospective study, 59 patients with treatment‐naïve autoimmune hepatitis were treated with prednisolone plus MMF, and 88% of patients had clinical and biochemical response.^28^ In another study, 29 patients with autoimmune hepatitis received MMF as first line (*n* = 17) or second line (*n* = 12) treatment; 34% of patients were intolerant to MMF, but the remission rate was 84%.^29^ These data may be interpreted as support for more frequent use of MMF for ICI‐related liver injury, but attention should be paid to the adverse events associated with MMF, such as headache, nausea, and cytopenia.^29^

Despite knowledge of ICI‐related hepatitis increasing, there is no consistent policy for tapering the immunosuppressive drugs after achieving remission in this condition. According to the guideline of the American Society of Clinical Oncology, corticosteroids can be tapered over 4–6 weeks in patients with grade 3/4 hepatitis.^9^ On the other hand, experienced clinicians sometimes taper steroids over a longer time and continue a maintenance dose of 5 mg/day.^30^ No uniform protocol for the tapering MMF has been established. In a clinical trial of autoimmune hepatitis, during a median follow‐up of more than 2 years, MMF was discontinued without relapse in only 5.1% of patients, whereas corticosteroids were discontinued in 57.6% of patients.^29^ Accordingly, we first reduced the dose of prednisolone while withholding MMF; in the future, we plan to taper MMF after discontinuing prednisolone. Further research is needed to determine the best practice for using MMF for ICI‐related liver injury. We also used ursodeoxycholic acid for both patients. Regarding autoimmune hepatitis, concomitant use of ursodeoxycholic acid of 300–600 mg/day may be effective to prevent early relapse during corticosteroids tapering^31^; however, it is unclear whether adding this drug is also effective for immune‐related liver injury.

Several radiological findings have been reported to be associated with immune‐related liver injury, including hepatomegaly, periportal edema, and periportal lymphadenopathy.^32^ Thickening of the gallbladder wall was also reported in a patient with ipilimumab‐induced hepatitis.^33^ In our experience, ultrasonography revealed thickened wall of the gallbladder in both patients, whereas CT and/or MRI did not show any abnormalities. Although more evidence will be needed to determine the diagnostic value of this finding for such patients, ultrasonography should be considered in patients suspected of immune‐related liver injury.

In conclusion, MMF appears to be an appropriate treatment option for hepatic irAEs that respond to high‐dose corticosteroids but relapse during steroid tapering.

## AUTHOR CONTRIBUTIONS

All authors were involved in diagnosis and treatment of the patients. *Conceptualization*, M.U. and H.T.; *Investigation*, M.U. and A.H.; *Formal Analysis*, M.U., H.T., T.K., Y.M., K.N. and M.M.; *Writing ‐ Original Draft*, M.U., H.T. and K.N.; *Writing ‐ Review & Editing*, A.H., T.K., Y.M. and M.M.; *Supervision*, K.N. and M.M.

## CONFLICT OF INTEREST

The authors have stated explicitly that there are no conflicts of interest in connection with this article.

## ETHICS STATEMENT AND CONSENT TO PARTICIPATE

Informed consent was obtained from the patients for publication of this case report. Following the protocol of the Medical Ethics Committee of Kurashiki Central Hospital, ethics review was not required.

## Data Availability

The data that support the findings of this study are available on request from the corresponding author. The data are not publicly available due to privacy or ethical restrictions.
